# Drawing on drawings: Moving beyond text in health professions education research

**DOI:** 10.1007/s40037-018-0436-7

**Published:** 2018-06-07

**Authors:** Charlotte Rees

**Affiliations:** Monash Centre for Scholarship in Health Education (MCSHE), Faculty of Medicine, Nursing & Health Sciences, Monash University, Melbourne, Australia

A Qualitative Space highlights research approaches that push readers and scholars deeper into qualitative methods and methodologies. Contributors to *A Qualitative Space *may: advance new ideas about qualitative methodologies, methods, and/or techniques; debate current and historical trends in qualitative research; craft and share nuanced reflections on how data collection methods should be revised or modified; reflect on the epistemological bases of qualitative research; or argue that some qualitative practices should end. Share your thoughts on Twitter using the hashtag: #aqualspace

In this edition of *Perspectives on Medical Education*, Kahlke & Eva employ visual elicitation to explore health educators’ understandings of critical thinking [1]. They first interviewed 16 educators about their conceptions of critical thinking in order to generate a researcher-produced mind map, which they then presented to study participants in their second interviews to encourage further elaboration on their understandings. While they argue that the mind map helped participants to provide sincere and unrehearsed responses, respond to others’ conceptualizations of critical thinking, and act as a member check, they acknowledge that their primary data were the interviews *not* the visual data. This privileging of talk and text over the visual is typical within social sciences research [2–5]. Therefore, this ‘*A Qualitative Space’* article serves to move beyond text to better foreground the visual in health professions education research (HPER). This article begins with a summary of visual methods, key philosophical issues, and important strengths and challenges. I then consider the use of visual methods in HPER, focusing specifically on drawings rather than videos as videos have been discussed recently elsewhere [6]. While drawings have received little attention in HPER, they are becoming increasingly employed [7–12]. I therefore showcase illustrative examples of HPER drawing on drawings: pre-existing, participant-produced and researcher-produced drawings in order to raise awareness of their potential for future HPER.

## An overview of visual methods

Visual methods can be described as: ‘the use of visual materials … employed by a social researcher during the course of an investigation’ [13, P. ix]. They reflect particular ways of ‘experiencing, expressing, sensing and … seeing’ [14, P. 29–30]. Visually-orientated disciplines are diverse including anthropology, sociology, cultural studies, psychology, history and geography [2, 5]. It is no surprise, therefore, that visual methods are also extremely heterogeneous including drawings, photography, graphic novels, video, and artworks such as clay modelling, collage, and quilting [2, 14, 15]. Visual materials can either be pre-existing (i. e. found) or researcher-instigated, either participant- or researcher-produced [5, 14, 16]. When researchers control graphics, speech is typically central to communication, whereas graphics are central to facilitating communication with participant-generated graphics [2]. Indeed, participant-generated visuals such as Photovoice typically emphasize participative modes of inquiry to give voice to those who are typically silenced [14]. Several visual scholars have argued that many analytical approaches employed for language (i. e. talk and text) can equally be applied to image-based research such as visual content analysis [3, 5, 16, 17]. However, other analytic approaches informed by visual and cultural studies can be derived from aesthestics [5]. Rose [18], for example, describes three intersecting sites (i. e. how the image is produced, what the visual effects are, and how the image is displayed to the audience) and three intersecting modalities (i. e. technological, compositional and social) for making sense of images [5, 17].

## Philosophical issues underpinning the visual

An ongoing philosophical question around the visual is the extent to which images *represent *or *construct* reality [14]. Indeed, “the image is never a ‘mere’ reflection of reality, but rather a multi-layered text …” [19, P. 45]. For early visual researchers such as 19th century anthropologists, researcher-produced images like photographs were felt to capture accurate and thus ‘truthful’ representations of reality [2]. The analytical focus then tended to centre on researchers’ understandings of the contents of images, with images often employed as adjuncts to text [2]. However, around the 1930s and onwards, social scientists began to problematize the objectivity of images [2]. Images are, for example, ambiguously polysemic, thus yielding multiple interpretations [4, 16, 19]. Visual researchers started instead to focus on broader considerations such as the social context in which images were created and how images were made sense of by researchers *and* participants [2]. From the 1980s, visual researchers moved towards multiple representations of the visual, researcher-found images, and considerations about how visual materials were perceived and experienced [2]. For example, considerations of the agency of images became important: What is the intention of the designer of an image? What does the image make us do? [3, 16]. Furthermore, what story does the image tell? [3, 16]. Researchers now go beyond the internal narrative (e. g. What is this image of?) to consider the external narrative (e. g. Who created the image and for whom? When and why was it created?) [3, 16]. Visual research methodologies therefore typically veer towards the exploratory rather than the confirmatory [16]. For some qualitative researchers, the processes of creating images (such as the use of participatory approaches) have become as important as the images themselves [14]. Furthermore, important questions arise about who is interpreting images and for what purposes, and how the interpretation of images interplays with the interpretation of text [14]. Researcher reflexivity is thus central with researchers thinking about and communicating their own roles within the visual research process [16].

## The benefits of visual methods

While we live in cultures of ocularcentrism [2, 16], visual research methods are often underutilized in the social sciences [4, 5, 19]. Visual methods have traditionally been reserved for visual topics (and often using single visual methods) but nowadays visually-orientated multi-method interdisciplinary approaches are becoming increasingly common in the social sciences [2, 4, 5]. Indeed, visual methods may be useful even when the topic of inquiry or research questions have no obvious visual components [2, 16]. Visual methods are often employed to enhance data collection through rapport building and empathy, facilitating communication, enhancing expression of tacit knowledge, accessing difficult to reach participants (e. g. those with low verbal literacy, children and so on), and encouraging reflection [2, 4, 14, 16, 20]. Indeed, many have argued that visual methods (and visual methods combined with text-based methods) can enable richer, more complex, deeper and sometimes hidden data to be collected [4, 6, 14, 17, 20, 21]. Furthermore, sometimes the visual can ‘disrupt well-rehearsed present narratives on a topic’ [4, P. 6], plus they can provoke and capture emotions, embodied states and spaces [2, 4]. Furthermore, focusing on images can enhance researcher observation and participants’ attention [2]. Visual methods have also been employed in order to change researcher-participant relationships with, for example, participant-generated images emphasizing participants as experts, shifting the balance of power away from researchers and towards participants, developing collaborative researcher-participant relationships and effecting change [4, 14, 15, 20]. Ultimately, visual research can counter the domination of text and counts in social sciences research [2].

## The challenges of visual methods

Visual methods are not without their challenges however. Most often discussed in the visual methods literature are issues pertaining to ethics and in particular informed consent and anonymity, plus data governance issues [2, 3, 6, 14, 20]. Informed consent is particularly problematic in the case of pre-existing and participant-generated visual materials. For example, individuals appearing in pre-existing materials such as ‘found’ photographs would not have given informed consent for their photographs to be used as research data. Furthermore, we cannot be sure that Photovoice participants have secured informed consent from all individuals appearing in the photographs they take and what does the researcher do if individuals have been photographed engaging in dangerous or illegal acts? [2, 22]. Indeed, visual images such as photographs and videos render research participants (and bystanders) more identifiable, making the maintenance of anonymity challenging, particularly when participants ask to remain anonymous. In terms of data governance, visual data alongside digital technologies trigger legal and cultural issues around the storing, retrieval and safety of data, making the overall management of data trickier than is the case with oral/written data [2, 6]. Other challenges discussed in the literature relate to the higher costs of visual methods such as equipment costs, but also the additional time-related costs of viewing and analyzing images [3, 16]. Finally, researchers often experience constraints in terms of presenting visual data in journal articles, either because of issues of anonymity (as above) or because print journals are reluctant to publish images (particularly colour images) because of increased print costs [16]. Indeed, while print-based journals might allow a few still images, they cannot publish video data [16]. Alternatively, online journals enable the publication of videos, with some journals now specializing in visual methods such as the Video Journal of Education and Pedagogy launched by Springer in 2009 (see: https://videoeducationjournal.springeropen.com/). Furthermore, although researchers often quote others’ words, they rarely cite others’ images [16].

## Drawings as an example of the visual

Despite these challenges, researchers are increasingly turning to visual methods in HPER, and in particular to researcher-generated video methods [6, 23, 24]. Less common in HPER is the use of photography [22], and drawings [21]. Drawings have been described as ‘the manual making of marks as a form of visual communication …’ [25, P. 41]. In the remainder of this paper, I concentrate on drawings as visual research methods in HPER for four reasons. First, to my knowledge, nobody has previously published a comprehensive discussion of a range of drawing-related visual methods in HPER (in contrast to previous discussions of video-based HPER) [6, 26]. Second, while drawing-based HPER is relatively uncommon, it is becoming increasingly popular [7–12]. Third, drawings are low-tech requiring only paper and pencil (in contrast to videos) [27]. And finally, manual drawing practices are widespread in healthcare practice and education [25].

So, what follows is two examples of HPER using pre-existing drawings as research data—comics and mind maps [7, 8], followed by two examples of HPER employing participant-produced drawings—Pictor technique and rich pictures [9, 10], and finally two examples of HPER employing researcher-produced drawings—visual notation and infographic visual representation of qualitative data [11, 12]. Note that I include visual representation of data here as qualitative researchers often do not present their textual data as images despite many scholars advocating the visual representation of data [2]. See Table 1 for a glossary of drawing terms. The remainder of this article presents a description of these diverse drawing methods alongside their strengths and challenges, in order to inspire you to consider the potential for drawings in HPER.

**Table 1:** Glossary of terms for types of drawings

*Comics*	Comics have been described as ‘graphic narratives’ or ‘graphic novels’, combining images with words to tell stories [7, 15]. Galman explained that ‘the comic book typically features the “muscled heroism of superheroes”, spectacular scenes, sound effects and exaggerated action’ [15, P. 200], thereby differentiating comics from ‘graphic novels’ (a sub-set of comics), which alternatively address political and autobiographical themes in more sedate and restrained ways
*Mind maps*	Like concept maps [28, 29], mind maps are: ‘graphic, schematic outcomes of learning activity, which aim to organize knowledge and clarify the learnt or investigated problem’ [8, P. 3]. Developed by Buzan & Buzan [30] mind maps are typically non-linear, tree-like structures with thick branches created from central concepts, moving to thinner branches for more peripheral concepts, and including more images than text [8]
*Pictor technique*	Originally developed in the context of family therapy [31], the Pictor technique involved giving clients blank arrow-shaped cards on which to write the names of family members and then to arrange the cards in ways reflecting how clients see family relationships [32]. The Pictor technique has been used more recently within the context of HPER employing interview-based methods to explore collaborative working, social support for medical educators and the impact of the clinical environment on students’ self-regulated learning [9, 32, 33]
*Rich pictures*	Rich pictures can be described as ‘pictorial representations that attempt to capture an individual’s perspective of a situation, including objects, ideas, people, character, feelings, conflicts and prejudices’ [34, P. 714–715]
*Visual notation*	Clare Kell developed a ‘novel notational system to capture, with paper and pencil in real time, the proxemics and kinesics of naturally occurring physiotherapy placement education interactions’ [35, P. 256]. The notation system involves the drawing of stick figures in real-time focusing on their use of space and movement (proxemics) and eye contact, gaze and paralanguage (kinesics) [36]
*Infographics*	The term infographics is a blend of the two words ‘information’ and ‘graphics’, so can be described as ‘information visualizations’ that are either the same or similar to existing forms of visual representations of data [37, P. 1]. They can be seen as a ‘knowledge assemblage’ including text, numbers, graphs, charts, drawings and so on [37]

### Pre-existing drawings as research data

**Fig. 1:**
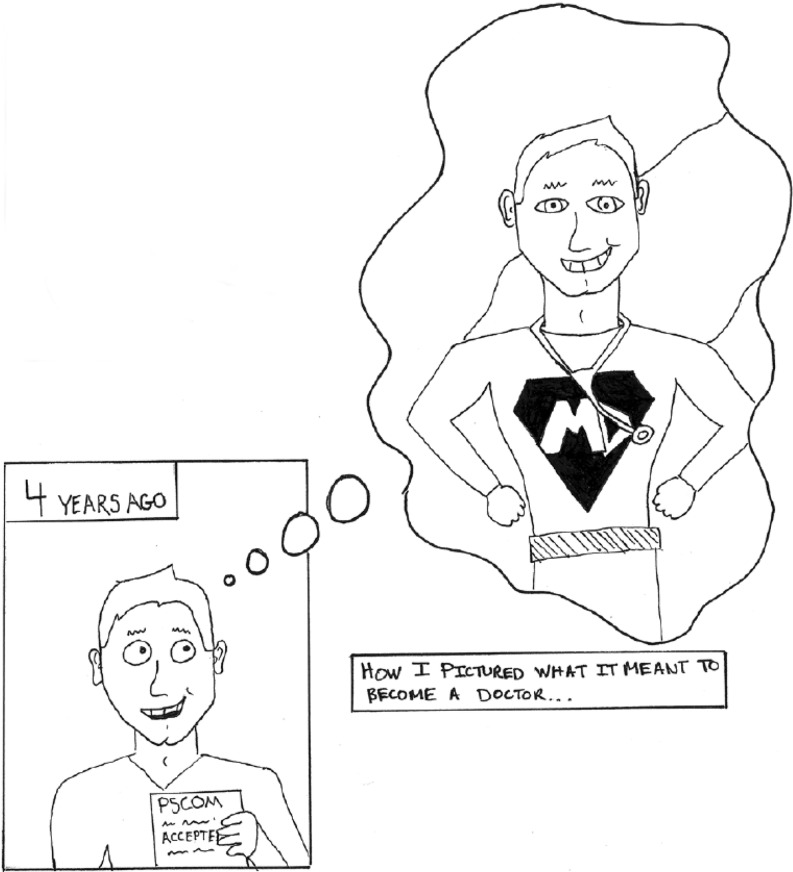
Pre-existing student comic from Green [7, P. 776] (reproduced with permission)

**Fig. 2:**
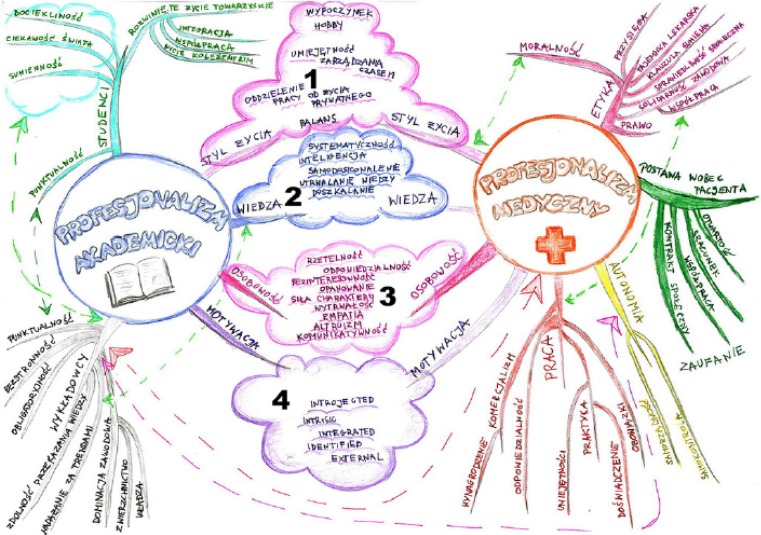
Pre-existing student mind map from Janczukowicz & Rees [8, P. 6] (reproduced with permission)

The comics and mind maps appearing in Green and Janczukowicz & Rees, respectively, can be described as pre-existing images because the drawings were collected through curricular interventions and then used later for research purposes [7, 8]. For example, Green conducted a thematic analysis of 58 medical students’ comics collected from the previous 6 years of a medical comics course in order to explore their professional identity formation (Fig. 1); [7]). Green identified five themes in medical students’ comics: how students found their place, medical students as patients, medical learning as transformation, relating to patients, and the joys and difficulties of becoming a doctor (Fig. 1; [7]). The strengths of comics as data included their power to: 1) facilitate students’ reflections on their professional identity development; 2) give voice to students’ concerns (and in ‘unfiltered’ ways) that may be difficult to put into words; and 3) *describe *and *show* how experiences influence students’ developing identities [7, 38]. Janczukowicz & Rees, on the other hand, conducted a semiotics-inspired framework analysis of 98 mind maps produced by 262 first-year medical students as part of their professionalism curricula to explore their understandings of academic and medical professionalism and their relationships (Fig. 2; [8]). They identified the most common textual and visual images associated with academic professionalism (text = learning, lifestyle and personality; image = books, academics caps and teachers) and medical professionalism (text = knowledge, ethics and patient-doctor relations; image = stethoscope, doctor and red cross) [8]. They found that while relationships between academic and medical professionalism were indicated in mind maps through visual connections and co-occurring text, students often struggled to visualize these relationships in their mind maps (Fig. 2; [8]). The strengths of mind maps as data included their ability to: 1) identify participants’ unrehearsed (and sometimes tacit) thinking and imagination about concepts and their relationships; and 2) present already visually structured data [8]. Given that both studies employ pre-existing images as data, however, neither engaged participants in discussions about their drawings: indeed, interpretations of the comics and mind maps were based on researcher analyses only. As discussed above, images are polysemic with multiple interpretations [4, 16, 19], and therefore many scholars suggest that researchers should collaborate with participants in the interpretation of images [15, 39].

### Participant-produced drawings as research data

**Fig. 3:**
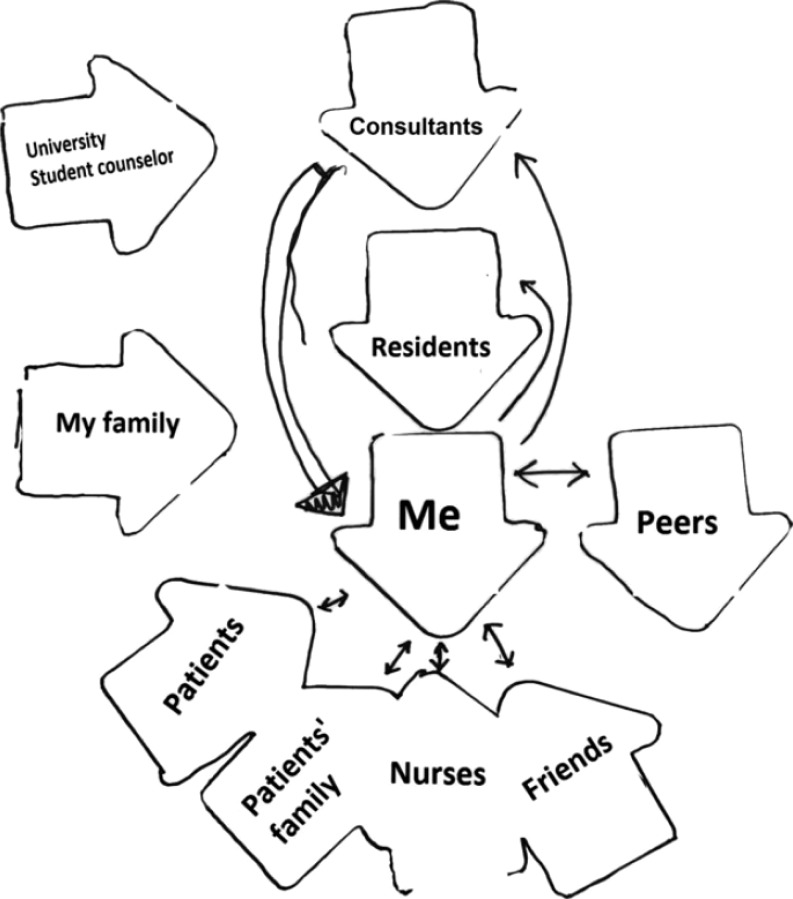
Participant Pictor chart from Berkhout et al. [9, P 273] (reproduced with permission)

**Fig. 4:**
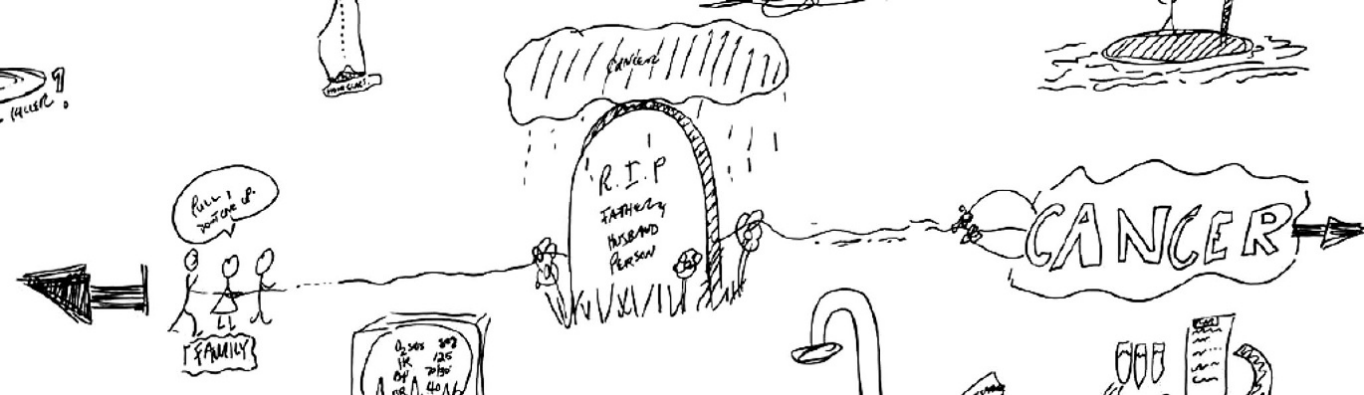
Participant rich picture from Cristancho et al. [10, P. 1543] (reproduced with permission)

The Pictor technique and rich pictures employed by Berkhout et al. and Cristancho et al. respectively can be seen as participant-produced drawings [9, 10]. Berkhout et al. employed the Pictor technique as part of 14 semi-structured interviews with medical students to explore who, how and to what extent people in the clinical environment influence students’ self-regulated learning (Fig. 3; [9]). They analyzed both the Pictor charts and interview transcripts using a grounded theory constant comparison and open, axial and interpretive coding [9]. They found that various people (e. g. peers, supervisors, hospital staff) influenced students’ self-regulated learning through learning opportunities, goal setting, self-reflection, role clarification and coping with emotions [9]. Differences existed between novice and experienced students in terms of others’ roles in self-regulated learning [9]. The Pictor charts highlighted positive and negative influences, power differences and barriers in terms of relationships (Fig. 3; [9]). The strengths of the Pictor technique include: 1) sensitizing participants to people (and all people) within complex settings thus stimulating recall; 2) facilitating participants’ storytelling through words *and *visuals; and 3) helping to visibilize intangible aspects of the topic of inquiry [9, 33]. Cristancho et al., on the other hand, utilized rich pictures collected as part of final interviews with five surgeons in order to better understand the complexities of challenging surgical situations (Fig. 4; [10]). They conducted an aesthetic analysis of each drawing, followed by a comparative analysis of multiple drawings and finally, a team-based analysis (involving participants) of the drawings [10]. They found that while interviews privileged the procedural dimensions of surgeons’ perspectives on complex and challenging operations, the drawings mostly underscored the non-procedural dimensions of challenging operations such as team dynamics, trust, emotions and external pressures (Fig. 4; [10]). The strengths of rich pictures as data include: 1) facilitating participants’ reflections on the complexity of situations; and 2) gaining insights into the unspoken/hidden aspects of the topic of inquiry [10, 21, 34, 40]. While Pictor charts have been criticized for being over-simplistic and restrictive because participants might prefer to use shapes other than arrows and represent the nature of relationships differently [32], rich pictures might suffer from being overly-complicated and therefore difficult and laborious to analyse [10].

### Researcher-produced drawings

**Fig. 5:**
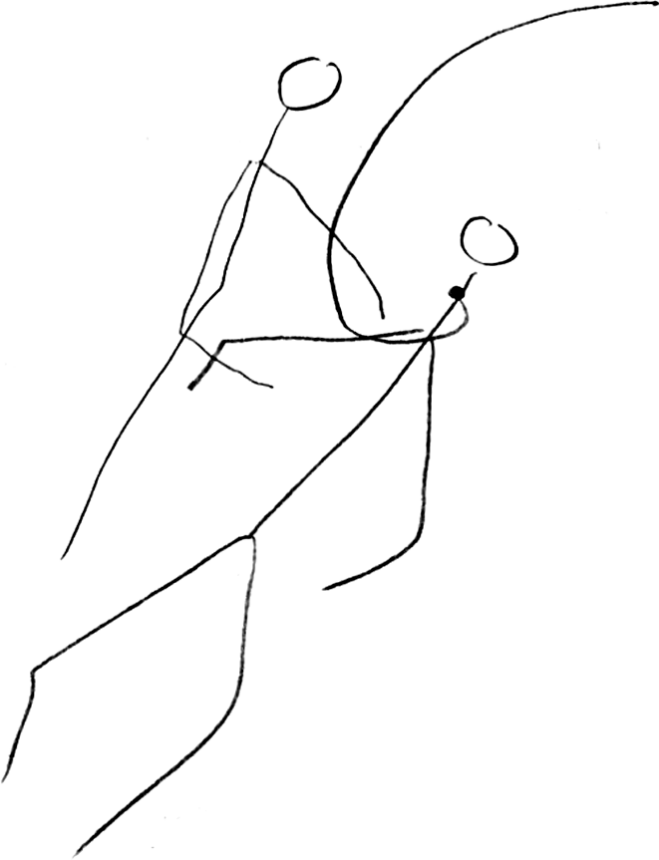
Researcher-generated visual notation of an experienced therapist helping a student identify physiotherapy work in the care of a semi-conscious patient from Kell [42, P. 363]

**Fig. 6:**
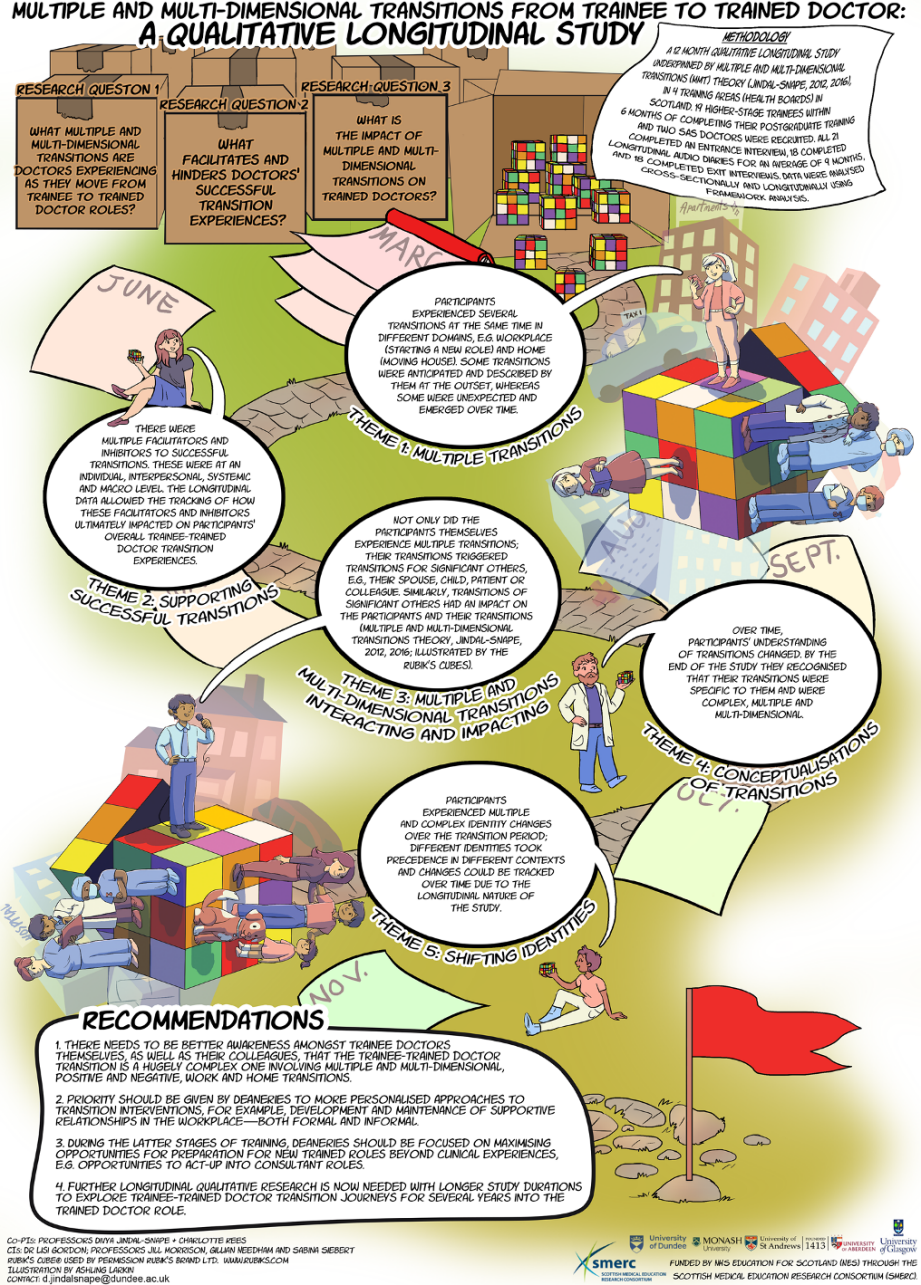
Researcher-generated infographic of findings for Gordon et al. [12, 41] (reproduced with permission)

Finally, I present two examples of researcher-produced drawings to illustrate how researcher-generated drawings can act as research data or be used to visually represent qualitative study findings [11, 12, 41, 42]. In terms of drawings as data, Kell employed her visual notation system (see Table 1) as part of an ethnography of clinical placements involving six final-year physiotherapy students to explore the extent to which patient-centred care involving students was enacted (Fig. 5; [42]). Kell analyzed the ‘what’s’ and ‘how’s’ of her observational data [43]. Data were first analyzed *within* context and then analyzed *across* contexts making visible educational practices [11]. Kell found that patients were typically absent in everyday placement activities: staff either practised skills on each other or discussed patients without patients present or patients were often objectified as teaching materials when they were present [11]. The strengths of Kell’s visual notation method is that it: 1) enables the quick real-time documentation (and thus visibilization) of observational data in sites disallowing recording devices such as video; and 2) facilitates the researcher’s close noticing of the minutiae of non-verbal practice [11, 27, 35, 36]. While all ethnographic researchers, irrespective of their recording methods, make real-time choices about what to record, video allows for more thoughtful considerations about the focus of analysis i. e. after the recording of events. Furthermore, given that Kell’s visual notation is based on the researcher’s real-time stick drawings, the recordable moments are inevitably more partial than those recorded with video [35, 36]. Finally, with respect to visual representations of qualitative data, Jindal-Snape et al. worked with a professional artist to produce an infographic to visually represent the themes identified in their qualitative longitudinal study with 19 higher-stage trainees exploring transitions into the trained doctor role (Fig. 6; [12, 41]). While the study infographic was not published in the journal article, it was disseminated through various channels including: 1) the ‘Dundee Comics Creative Space’ event at the University of Dundee in December 2017 (see: https://www.dundee.ac.uk/tcelt/news/2018/articles/comic-and-infographic-launch-.php); 2) the web such as the funder’s website (http://www.scotlanddeanery.nhs.scot/media/80214/transitions-infographic-2017.jpg), the website for the CORE open access publisher (https://core.ac.uk/download/pdf/141204361.pdf) and a research network’s blog (https://educationalandlifetransitionsresearchandpractice.wordpress.com); and 3) through social media such as Twitter. The researchers designed the infographic to create what they thought would be a powerful visual message simplifying the complexity of their study findings to more successfully disseminate their findings to the end-users of the research, namely doctors, policy-makers, training organizations, and institutional leaders [5, 37].

## Summary and conclusions

To conclude, visual methods are increasingly used in HPER including the use of pre-existing, participant-produced and researcher-generated drawings. Key benefits of drawings in HPER so far have included: facilitating participants’ thinking, recall and storytelling about complex topics of inquiry [7–10]; eliciting data from participants that is both unrehearsed and hard to get at using alternative methods such as interviews [7–10, 21]; enabling the documentation of observational data when recording devices like video are not permissible [27]; enhancing the visual capabilities of researchers [11]; and communicating the findings of qualitative research to end-users in powerful ways [5, 37]. While Prosser & Loxley have suggested that: “Visual methods are difficult and complex … becoming a more ‘seeing’ researcher is not an easy option” [2], I hope this *‘A Qualitative Space’* paper has illustrated how some HPE researchers are using visual methods and thus becoming more ‘seeing’. Of course, visual methods should never be used as an end in themselves, just because they are innovative or fun, but rather they should be employed as a means to an end: to help answer serious research questions underpinning inquiry [3, 16]. I would thus recommend the thoughtful and appropriate use of visual methods as part of multi-method interdisciplinary approaches [2, 4, 5, 16]. I therefore invite you to consider drawing on drawings in your own research in order to move beyond text.
